# Can supernatant from immortalized adipose tissue MSC replace cell therapy? An in vitro study in chronic wounds model

**DOI:** 10.1186/s13287-020-1558-5

**Published:** 2020-01-21

**Authors:** Honorata Kraskiewicz, Maria Paprocka, Aleksandra Bielawska-Pohl, Agnieszka Krawczenko, Kinga Panek, Judyta Kaczyńska, Agnieszka Szyposzyńska, Mateusz Psurski, Piotr Kuropka, Aleksandra Klimczak

**Affiliations:** 1 Laboratory of Biology of Stem and Neoplastic Cells, Hirszfeld Institute of Immunology and Experimental Therapy, Polish Academy of Sciences, ul. Rudolfa Weigla 12, 53-114 Wroclaw, Poland; 2 Department of Histology and Embriology, Wroclaw University of Environmental and Life Sciences, ul. Norwida 31, 50-375 Wroclaw, Poland

**Keywords:** Adipose tissue-derived mesenchymal stem cell, Stem cell supernatants, Chronic wound

## Abstract

**Background:**

Mesenchymal stem cells (MSCs) secrete a cocktail of growth factors and cytokines, which could promote tissue regeneration and wound healing. Therefore, in clinical practice, post-culture MSC supernatant treatment could be a more attractive alternative to autologous stem cell transplantation. In this study, we compared the regenerative properties of supernatants harvested from four newly established human adipose tissue mesenchymal stem cell lines (HATMSCs) derived from chronic wound patients or healthy donors.

**Methods:**

HATMSC supernatants were produced in a serum-free medium under hypoxia and their content was analyzed by a human angiogenesis antibody array. The regenerative effect of HATMSCs supernatants was investigated in an in vitro model of chronic wound, where cells originating from human skin, such as microvascular endothelial cells (HSkMEC.2), keratinocytes (HaCaT), and fibroblasts (MSU-1.1), were cultured in serum-free and oxygen-reduced conditions. The effect of supernatant treatment was evaluated using an MTT assay and light microscopy. In addition, fibroblasts and HATMSCs were labeled with PKH67 and PKH26 dye, respectively, and the effect of supernatant treatment was compared to that obtained when fibroblasts and HATMSCs were co-cultured, using flow cytometry and fluorescent microscopy.

**Results:**

A wide panel of angiogenesis-associated cytokines such as angiogenin, growth-regulated oncogene (GRO), interleukin-6 and 8 (IL-6, IL-8), vascular endothelial growth factor (VEGF), insulin growth factor 1 (IGF-1), and matrix metalloproteinase (MMP) were found in all tested HATMSCs supernatants**.** Moreover, supernatant treatment significantly enhanced the survival of fibroblasts, endothelial cells, and keratinocytes in our chronic wound model in vitro. Importantly, we have shown that in in vitro settings, HATMSC supernatant treatment results in superior fibroblast proliferation than in the case of co-culture with HATMSCs.

**Conclusions:**

Our results suggest that therapy based on bioactive factors released by the immortalized atMSC into supernatant has important effect on skin-derived cell proliferation and might preclude the need for a more expensive and difficult cell therapy approach to improve chronic wound healing.

## Background

Chronic wound patients have a reduced ability to undergo normal tissue repair and are more susceptible to ulcers, resulting in patient trauma, and often the need for limb amputation [1, 2]. At present, chronic wounds impact four million Europeans per year, and at least 1% of people living in high economy countries will experience a complex wound in their lifetime [3, 4]. Existing therapies for chronic wounds are expensive, and approximately 2–4% of the total European health budget is being spent on wound care [5]. Because of population aging and the increasing incidence of diabetes, these costs are expected to increase dramatically in the near future. One of the most promising approaches to wound healing, due to enhancement of the regenerative processes, is adipose tissue-derived mesenchymal stem cell (atMSC)-based therapy [6, 7]. For autologous cell transplantation, from all tissue sources of MSC adipose tissue is one of the most accessible. MSCs themselves possess high self-renewal potential, differentiation capacity, and secretory properties, thus, making them useful for clinical application [8]. According to the world’s largest clinical trials database run by the US National Library of Medicine at the National Institutes of Health, five clinical studies are currently ongoing to test the potential of atMSCs in the treatment of chronic wounds associated with diabetic foot syndrome or venous stasis ulcer (https://clinicaltrials.gov). However, the therapy itself is not efficient enough [9, 10]. This might be due to the fact that the survival of transplanted cells is limited, and their fate and function are not fully understood. Moreover, a sub-population of patients does not qualify for the treatment due to advanced age, co-morbidities, or reduced adipose tissue deposition.

It is known that atMSCs secrete a cocktail of growth factors and cytokines, which are involved in the main phases and events of the wound-healing process, such as induction of angiogenesis, reduction of inflammation, promotion of cell migration and differentiation, and collagen production and remodeling [11, 12]. In particular, qualitative analysis of MSC-conditioned medium revealed the presence of many growth factors, including epidermal growth factor (EGF), which plays a significant role in re-epithelization of skin wounds; fibroblast growth factor (FGF), supporting cell survival; insulin-like growth factor (IGF), which is involved in wound closure; and vascular endothelial growth factor (VEGF), a major regulator of angiogenesis [13]. Immunomodulatory cytokines such as IL-6 or IL-8 were also reported to be present in stem cell secretomes and had a proven role in angiogenesis and anti-inflammatory activity [14]. Finally, inflammatory regulatory chemokines such as macrophage chemoattractant protein-1 (MCP-1), matrix metalloproteinase (MMP), and tissue inhibitor of metalloproteinase (TIMP), which are involved in tissue remodeling, were also found in MSC-conditioned medium [13, 15]. Therefore, application to the wound of MSC-conditioned medium containing these biologically active molecules instead of whole cells seems to be a safer and cheaper approach. To address issues related to cell transplantation therapy and at the same having in mind the potential for implementation, we first present here the development and characterization of human adipose tissue mesenchymal stem cell lines (HATMSCs) that have high rates of proliferation and the capacity to secrete potent regenerating factors. Next, using an in vitro model of chronic wound, we compared regenerative properties of supernatants harvested from HATMSCs derived from a chronic wound patient to those generated from healthy donor adipose tissue. This study tests the hypothesis that an immortalized human atMSCs produce bioactive factors which promote proliferation and survival of human skin origin cells subjected to in vitro model of chronic wound.

## Methods

All reagents used in this study were purchased from Sigma-Aldrich, Poznan, Poland Ltd., unless otherwise stated. All tissue culture materials were purchased from BD Biosciences, Warsaw, Poland, unless otherwise stated.

### Human adipose tissue and atMSC isolation

Primary atMSCs were isolated from abdominal area adipose tissue derived from a patient suffering from venous stasis ulcer (82 years old) and a healthy donor (22 years old) from The Regional Specialist Hospital in Wroclaw, Poland. Cell isolation was carried out as part of the WROVASC project. Cells were isolated from the lipoaspirate, with a closed and fully automated CELLUTION 800 system (Cytori Therapeutics, San Diego, USA), using a dedicated enzymatic preparation Celase. The study protocol was approved by the local bioethics committee at the Regional Specialist Hospital, Research and Development Centre in Wroclaw (No. KB/27/2015). Informed consents of the patients were obtained.

### Primary atMSC cell transfection

In order to generate stable cell lines, primary atMSCs were transfected respectively with pSV3-neo and hTERT plasmids using ViaFect™ Transfection Reagent (Promega, Mannheim, Germany) according to the manufacturer’s protocol. Briefly, 1 day before transfection, cells were seeded in the 24-well plate (2.0 × 10^4^ cells/well) in Opti-MEM with GlutaMAX (Thermo Fisher Scientific Inc., Warsaw, Poland) with 3% human serum (HS) (Regional Centre for Blood Donation and Treatment in Wroclaw). Two micrograms of DNA plasmids were mixed with 200 μl of Opti-MEM medium and 6 μl of ViaFect™ transfection reagent. Following 20 min incubation at room temperature (RT), DNA-Transfection Reagent complex was mixed with 2 ml of serum-free Opti-MEM medium and 0.5 ml of the mixture was applied to one well of a 24-well plate. After 24 h incubation at 37 °C, 5% CO_2_, 0.5 ml of 3% HS Opti-MEM medium was applied to each well, while the whole medium was exchanged following 48 h.

### Selection of transfected cells

In order to distinguish non-transfected cells from those that have taken up the exogenous DNA, a drug selection with Geneticin and Puromycin for pSV3-neo and hTERT plasmids, respectively, was performed. Briefly, cells were cultured in nonselective medium (DMEM, 10% HS, 1% L glutamine, 1% penicillin/streptomycin (pen/strep)) for 2 days post-transfection and then switched to selection medium (with addition of 0.5 μg/ml Puromycin or 50 μg/ml Geneticin). As an antibiotic control, non-transfected cells were used. The use of selection medium was continued for 3 weeks, while the dose of Puromycin and Geneticin was gradually increased to the final concentrations of 10 μg/ml and 1000 μg/ml, respectively. Significant toxicity (> 90%) of non-transfected cells was observed following 4–5 days of incubation with the low dose of selecting drugs, while positively transfected cells were viable even at the maximum dose. Immortalized atMSCs derived from the venous stasis ulcer patient were named the HATMSC1 cell line, whereas immortalized atMSC derived from a healthy donor were named the HATMSC2 cell line.

### Single-cell cloning by serial dilution

To obtain a clonal population which consists of genetically identical cells, a serial dilution method was used. The method was performed according to the cell cloning by serial dilution in 96-well plates protocol. Briefly, 200 μl of HATMSC2 cell suspension at 5.0 × 10^4^ cells/ml were placed in A1 well of a 96-well plate and then 1:2 series dilutions were done down the entire column. Then, using a multi-channel pipette, 1:2 dilutions were done across the entire plate. The plate was incubated at 37 °C, 5% CO_2_ for 7 days and wells were monitored under the inverted light microscope to detect single clones. Several clones were visible under the microscope on day 2; however, only clones detected in well D10 (HATMSC2D10) and F10 (HATMSC2F10) demonstrated high proliferation rate and therefore were selected for further cell expansion.

### Immunophenotypic characterization

Cultures of HATMSC1, HATMSC2, HATMSC2D10, and HATMSC2F10 were phenotypically characterized by flow cytometry (FC) using phycoerythrin (PE)-tagged antibodies: CD73, CD90, CD105, CD146, CD34, CD45, HLA-ABC, and HLA DR (BD Biosciences, San Jose, USA). Antibody labeling was performed according to the manufacturer’s instructions. Briefly, 5.0 × 10^5^ cells were resuspended in 50 μl of 3% FBS in PBS buffer containing 3 μl of fluorescently labeled antibody. Following a 30 min incubation at RT in dark, cells were washed to remove non-conjugated antibodies and analyzed by flow cytometry (FC) using FACSCalibur (Becton Dickinson, San Joes, USA). Expression levels of selected antigens were evaluated with CellQuest software (Becton Dickinson, San Jose, USA).

### Tumorigenicity assay in NOD SCID mice

The established HATMSC1 and HATMSC2 cells were evaluated for potential tumorigenicity using NOD/SCID female mice (NOD.CB17-Prkdcscid/J strain). Cells suspended in PBS were subcutaneously injected into right body flank (10^7^ cells/mouse; one injection per animal). During the next 16 weeks, mice were monitored daily for body weight changes as well as for any signs of tumorigenicity (palpably tumors, skin changes at the place of injection). By the end of the experiment, mice were sacrificed, and the main organs (liver, spleen, kidney) and skin from the vicinity of the injection site were fixed in formalin and subjected to histopathological analysis. The experiments were performed according to Interdisciplinary Principles and Guidelines for the Use of Animals in Research, Marketing and Education issued by the New York Academy of Sciences’ Ad Hoc Committee on Animal Research and were approved by the Local Committee for Experiments with the Use of Laboratory Animals, Wroclaw, Poland (permission no. 47/2018).

### HATMSC supernatant production

Primary HATMSC2 were seeded in cell dishes (6 cm) at the density of 1.9 × 10^4^ cells/cm^2^ in DMEM, 10% HS, while HATMSCs were seeded in a three-layer T175 flasks (Thermo Scientific, Roskilde, Denmark) at the density of 1.9 × 10^4^ cells/cm^2^ in DMEM, 10% HS. Following 24 h incubation at 37 °C, 5% CO_2_, the culture medium was removed from the flask, and culture dish was washed and replaced with DMEM without serum. Following 24 h culture under hypoxic conditions (1% O_2_, 5% CO_2_), conditioned medium was collected and centrifuged for 10 min, 300 g to remove cellular debris. Collected native HATMSCs supernatants were store at − 20 °C or were concentrated with Amicon® Ultra 15 ml centrifugal 3 kDa filters (Merck Millipore, Carrigtwohill, Ireland) which resulted in approximately a 10-fold (v/v) enrichment. Total protein concentration in the native and concentrated supernatants was measured based on the method of Bradford using a Bio-Rad Protein Assay (Bio-Rad, Munich, Germany).

### Evaluation of the secretion profile of HATMSCs

Semi-quantitative detection of cytokines secreted by HATMSC to the supernatants was done with the use of the C-Series Human Angiogenesis Antibody Array (RayBio®, Norcross, USA) according to the manufacturer’s instructions. Briefly, 2 ml of blocking buffer were applied on the membrane and incubated 30 min at room temperature. Then 1 ml of HATMSCs supernatant was incubated with a membrane overnight at 4 °C. Following a series of washes, a biotinylated antibody cocktail was applied on the membrane and incubated for 2 h at room temperature. Unbound antibody was removed by series of washes, and the membrane was placed in HRP-Streptavidin and incubated for 2 h at room temperature. Following a third series of washes, chemiluminescence detection was performed and bound proteins were then visualized using X-ray film. A comparison of signal intensities was performed using ImageJ software (MosaicJ, Philippe Thevenaz) where relative differences in expression levels of each analyzed sample was measured using the Protein Array Analyzer plugin. Automatic analysis of obtained data was calculated using Microsoft® Excel-based Analysis Software Tool for Human Angiogenesis kit. To validate semi-quantitative results of the cytokines’ presence in the supernatants on the heat map, several cytokines such as EGF, FGF-2, GRO, MCP-1, Regulated on Activation, Normal T Cell Expressed and Secreted (RANTES), and VEGF were also detected using Human Cytokine/Chemokine Magnetic Bead Panel MILLIPLEX® MAP Kit (Merck, Darmstadt, Germany) according to the manufacturer’s instructions with standards and samples in duplicate, overnight incubation with shaking at 4 °C (18 h, 750 rpm), and using a hand-held magnetic block for wash steps. Data were acquired on a validated and calibrated MAGPIX® system (Luminex) with xPONENT® software. The median fluorescence intensity (MFI) of standards, control, and samples was measured and analyzed in Milliplex Analyst software using a five-parameter logistic curve-fitting method for calculating cytokine concentrations in samples.

### In vitro model of chronic wound

Normal human skin microvascular endothelial cells (HSkMEC.2) obtained and patented by our research group in cooperation with C. Kieda from Centre National de la Recherche Scientifique, France (patent 99–16,169), were cultured in Opti-MEM with GlutaMAX supplemented with 2% fetal bovine serum (FBS, HyClone, UK) and 1% pen/strep. The human keratinocytes cell line (HaCaT) was purchased from the DKFZ collection [16]. HaCaT cells and fibroblast cell line MSU-1.1 (kindly gifted from C. Grillon from Centre National de la Recherche Scientifique, France) [17] were cultured in DMEM, 10% FBS, and 1% pen/strep. To mimic the chronic wound microenvironment, skin cells were cultured under hypoxic conditions (1% O_2_, 5% CO_2_) in DMEM serum-free medium. Cells were seeded in 96-well plates at the density 2000 cells per well and treated with several doses of native (10, 25, 50, and 100%) or concentrated (1, 5, 10, and 15 μg) HATMSC supernatants. Metabolic activity (MTT assay) was measured at time 0 and following 24, 48, and 72 h. Non-treated cells were used as controls. For supernatant collected from primary HATMSC2, cell culture proliferation activity was measured only with MSU-1.1 cells in a concentration of 50% native supernatant.

Migration activity of MSU-1.1 fibroblast cell line was analyzed in a wound-healing assay. The cells were seeded in a 48-well plate at a density of 4 × 10^4^ in DMEM with 10% FBS. After 24 h of culture in standard conditions (37 °C, 21% O_2_, 5% CO_2_) when cells reached 100% of confluence, scratches were made with a sterile 200-μl pipette tip. The culture medium was then immediately removed (along with any dislodged cells). The removed medium was replaced with a fresh serum-free culture medium (control) or culture medium containing 50% native supernatants collected from HATMSC1, primary HATMSC2 cells, HATMSC2, HATMSC2D10, and HATMSC2F10 cells. All scratch assays were performed in duplicate.

Cell migration activity was investigated at 37 °C in an incubation chamber (PeCon GmbH, Erbach, Germany) with 1% O_2_, 5% CO_2_ mounted on an Axio Observer inverted microscope equipped with a dry 5× objective (Zeiss, Gottingen, Germany). The movement of the cells was time-lapse recorded for 44 h at intervals of 2 h using Zen 2.6 Blue Edition Software (Zeiss, Gottingen, Germany). Wound closure was analyzed using Zen Blue Software. Relative wound closure (RWC) was calculated as previously described [18].

### In vitro angiogenesis

The pro-angiogenic potential of HATMSC supernatants and primary HATMSC2 cells was tested by the tube formation assay using growth factor-reduced Matrigel (BD Bioscience, Bedford, USA). HSkMEC.2 cells were seeded into a Matrigel-coated 96-well plate at a density of 1.5 × 10^4^ cells per well. Cells were treated with 10 and 50% HATMSC supernatants in serum-free DMEM, and then cell cultures were monitored under the inverted light microscope every 2 h, until pseudovessels were completely formed. Non-treated cells were used as controls. Angiogenesis was calculated using the ImageJ Angiogenesis Analyzer software, by measurement of mean mesh size, number of meshes, number of nodes, and total length of the tubes.

### Cell fluorescent labeling and co-culture

MSU-1.1 and HATMSC2 cells were labeled with PKH67 (green) and PKH26 (red) dyes, respectively, according to the manufacturer’s recommendation. Labeled cells were seeded at a density of 3 × 10^4^ cells/well on six-well plates as follows: HATMSC2 alone, MSU-1.1 alone, co-culture of MSU-1.1 with HATMSC2 cells, and MSU-1.1 treated with 15 μg/100 μl of concentrated supernatant collected from HATMSC2 cells. The co-culture group consisted of 3 × 10^4^ MSU-1.1 and 3 × 10^4^ HATMSC2s. Cells were subjected to the in vitro chronic wound model (serum-free DMEM, 1% O_2_) for 1, 2, and 3 days. At each time point, the total number of cells and the average intensity of fluorescence were analyzed using a flow cytometer FACSCalibur (BD Bioscienses, San Jose, USA). Briefly, cells were trypsinized, centrifuged, and cell pellets resuspended in 200 μL of PBS before incubating with 20 μl of CountBright™ absolute counting beads (Thermo Fisher, Oregon, USA). To evaluate the number of cells in each group, 10,000 counting beads were collected using BD CellQuest™ acquisition software (BD Bioscience, Bedford, USA). Data analysis was performed using Flowing Software 2.0 (Perttu Terhoa). In addition, on the third day of the experiment, pictures of representative fields from each experimental group were taken using an Axio Observer inverted microscope (Zeiss, Gottingen, Germany).

### Statistical analysis

All statistical analyses were performed using GraphPad Prism version 7 (GraphPad Software, Inc.). Data were compared using a one-way analysis of variance (ANOVA) followed by multiple comparison procedures (Dunnet’s test). Values were considered as significantly different when *p* values were < 0.05.

## Results

### Immortalized HATMSC cell lines express typical mesenchymal markers

**Fig. 1:**
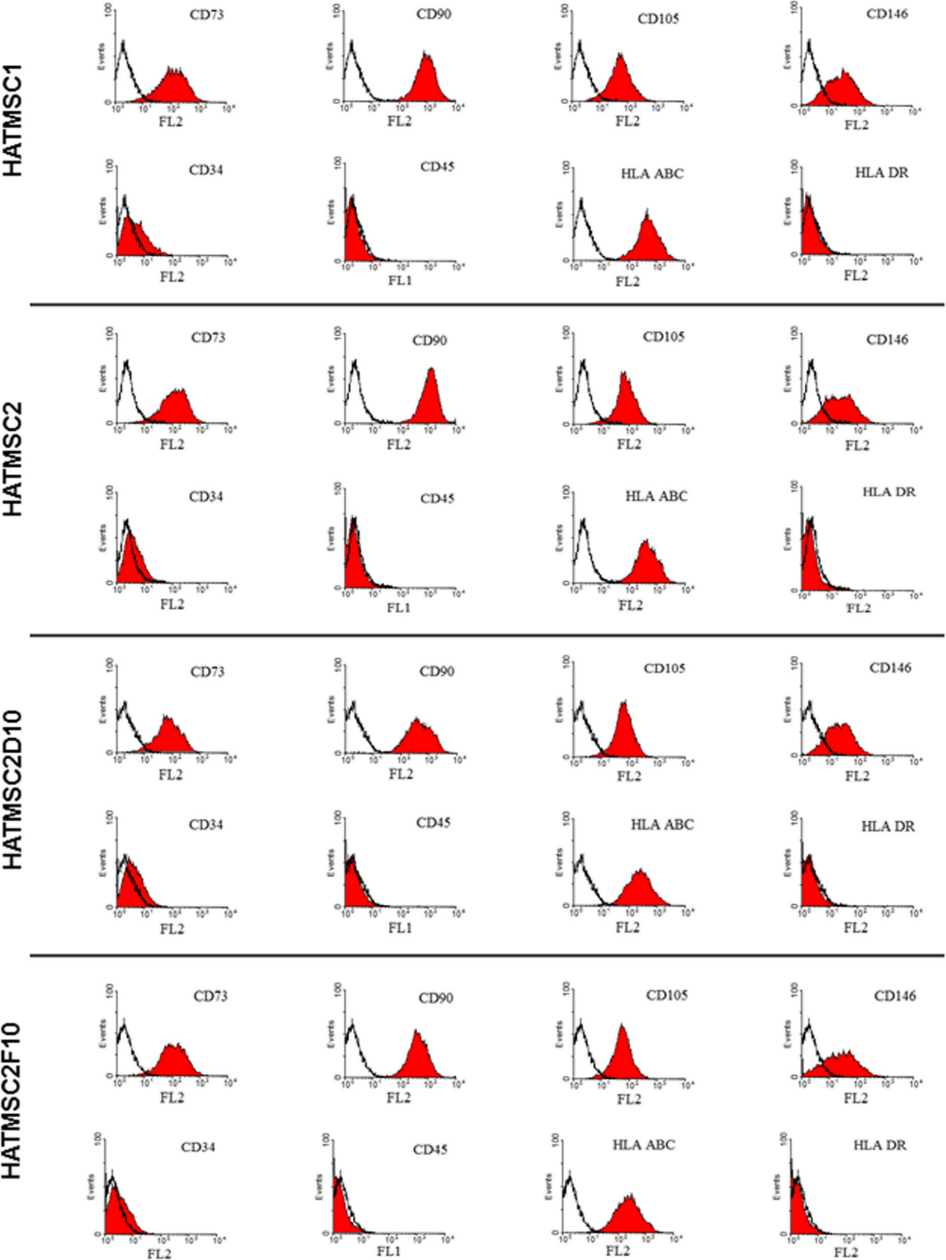
Phenotypic characterization of the HATMSC cell lines. The mean fluorescent intensity of HATMSC1, HATMSC2, HATMSC2D10, and HATMSC2F10 cells was reported on the *x*-axis. Black line curves represent isotypic control; red fields represent fluorescent peaks

Following transfection with pSV3-neo and hTERT plasmids and subsequent antibiotic selection, phenotypic characterization of all four HATMSC cell lines was performed using flow cytometry. Figure 1 shows that all HATMSC cells are positive for markers of MSCs, i.e., CD73, CD90, CD105, CD146, CD45, and HLA-ABC antigens, and negative for HLA-DR and CD45. In addition, minimal expression of CD34 was observed. The above panel of cell surface antigens was analyzed several times in a time-course manner up to 12 months of cell culturing and no significant changes in the expression profile was observed.

### HATMSCs are non-tumorigenic

All four developed HATMSC cell lines proliferated equally well under normoxic (21% O_2_) or hypoxic (1% O_2_) conditions (Additional file 1). When implanted in vivo, no signs of tumorigenicity were observed and mouse body mass continuously increased as expected for a healthy animal. At days 12–15, some small, palpable nodules developed at the site of cell injection, but they never exceeded 2.0 mm^2^ (unmeasurable by a caliper), and after the next 7–10 days, they disappeared. During autopsy, no obvious signs of angiogenesis nor ongoing tumorigenicity was observed at the injection site. In addition, histopathological analysis of skin, subcutaneous tissue, lung, spleen, liver, and kidney confirmed in vivo observation where no evidence of a tumor was observed following the injection of HATMSC cells (Additional file 2). Similar results were seen in the case of subcutaneous injection of all HATMSC lines.

### Immortalized HATMSCs secrete a wide panel of angiogenesis-associated cytokines

**Fig. 2:**
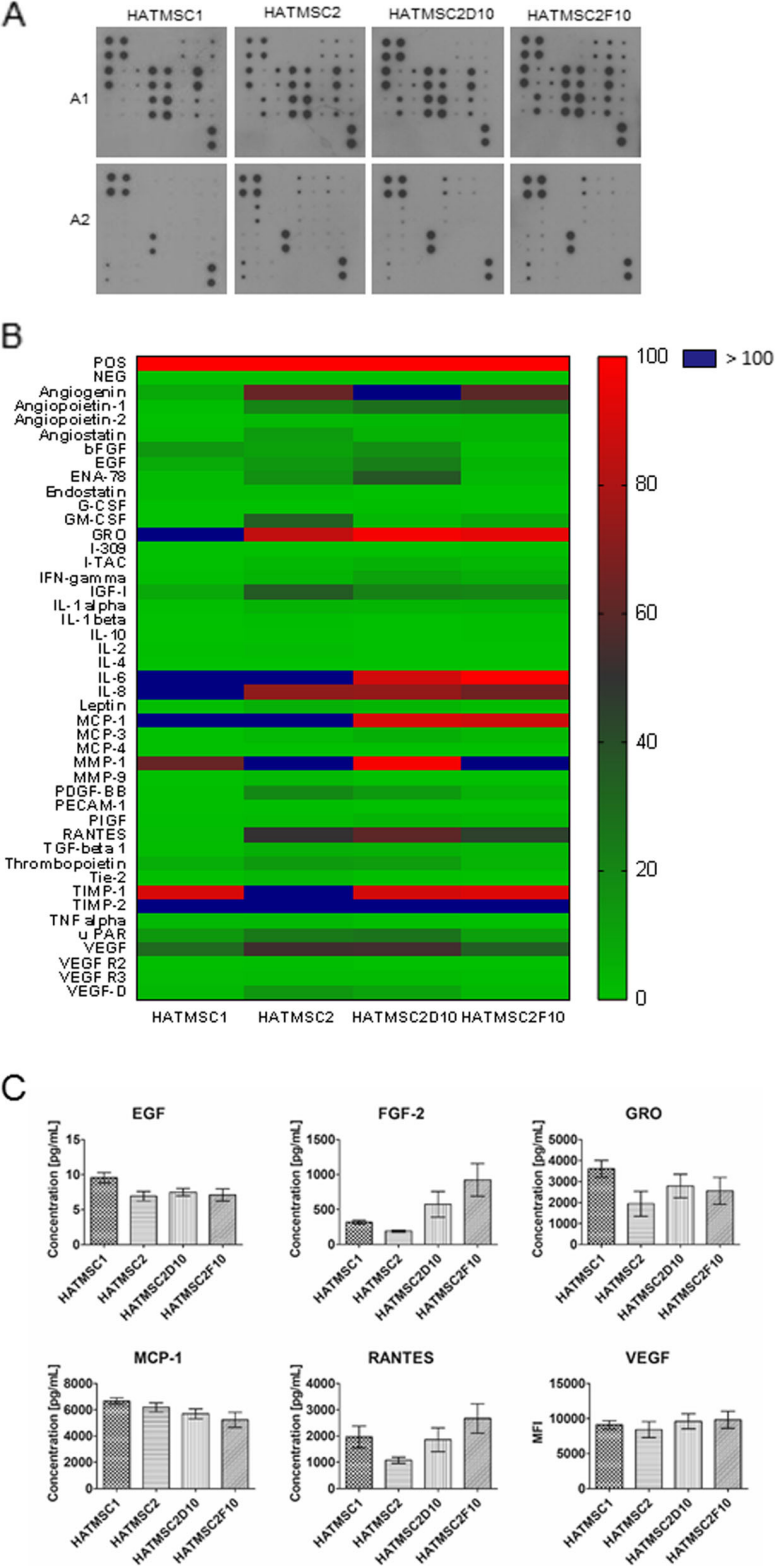
Production of angiogenesis-associated cytokines by HATMSC cell lines. **a** Scans of representative antibody arrays for HATMSCs supernatants. The signal intensity for each antibody spot is proportional to the relative concentration of the antigen in that sample. **b** Heat map of the cytokine data for HATMSCs supernatants. Data are normalized to the internal positive control spots which are consistent from array to array and represent 100%. Data represents mean, *n* = 2. **c** Concentrations of the selected cytokines found in all HATMSC supernatants measured by ELISA. For VEGF histogram, median fluorescence intensity (MFI) is presented on the *y*-axis since the detected concentration exceeded the maximum range of the standard curve. Data represents mean ± SD, *n* = 3

Analysis of the secretion profile of all four atMSC lines confirmed the presence of many biological factors involved in wound healing. From 43 human angiogenesis-related cytokines, 12 were found in all tested supernatants at the expression level ≥ 5% in comparison to the internal antibody array control. The most abundant secretion levels in all atMSC supernatants were detected in the case of pro-angiogenic cytokines such as angiogenin, growth-regulated oncogene (GRO), interleukin-6 and 8 (IL-6, IL-8), the chemokine RANTES, and vascular endothelial growth factor (VEGF). High levels of angiogenesis regulatory molecules such as insulin growth factor 1 (IGF-1), monocyte chemoattractant protein-1 (MCP-1), matrix metalloproteinase 1 (MMP-1), and tissue inhibitors of metalloproteinase 1 and 2 (TIMP-1 and 2) were also detected in all four supernatants. Validation of the selected factors by ELISA confirmed that all tested supernatants contain an abundant level of VEGF which exceeds the maximum range of the standard curve (> 10,000 pg/ml). In addition, a high concentration of MCP-1 (approximately 6000 pg/ml) was also found in all four supernatants. In contrast, EGF concentration in all supernatants ranged between 7 and 10 pg/ml. Differences between HATMSC supernatants were seen in the concentration of GRO, RANTES, and FGF-2. A summary of the investigated angiogenesis-related cytokines detected in atMSC supernatants is presented as a heat map in Fig. 2 a and b, while the concentration of the selected factors within supernatants are presented on histograms in Fig. 2 c.

### Native HATMSC supernatants significantly increase survival of human skin origin cells subjected to in vitro model of chronic wound

**Fig. 3:**
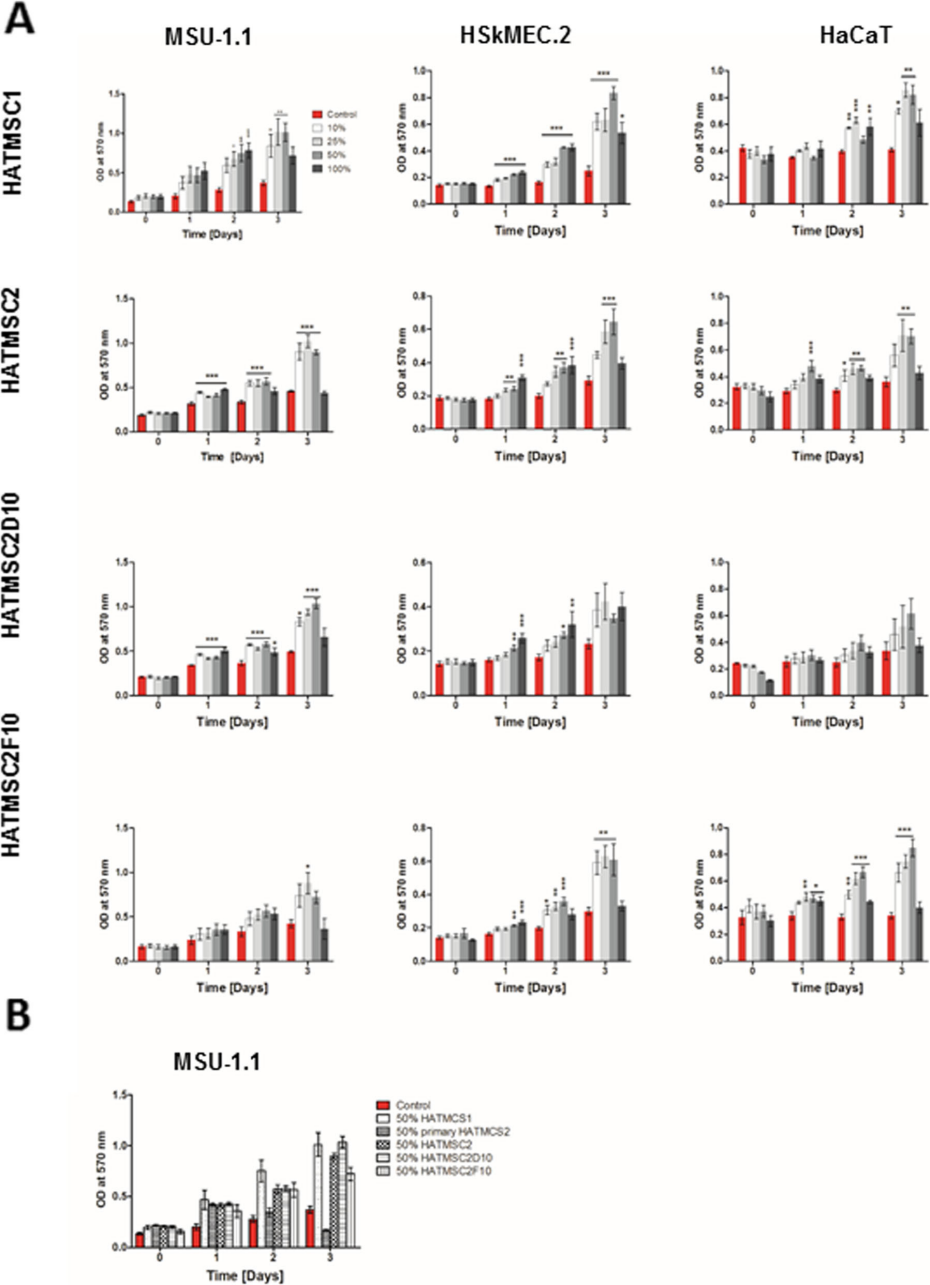
Proliferative activity of native HATMSC supernatants in in vitro model of the chronic wound. **a** Metabolic activity of MSU-1.1, HSkMEC.2, and HaCaT cells cultured in serum-free medium and 1% O_2_ was measured by MTT assay at days 0, 1, 2, and 3 following treatment with 10, 25, 50, and 100% of HATMSC supernatants. Untreated cells were used as a control. Data represents mean ± SEM, *n* = 3; *p < 0.05, **p < 0.01, ***p < 0.001. **b** Metabolic activity of MSU-1.1 cultured in the 50% supernatant from HATMSC1, primary HATMSC2, HATMSC2, HATMSC2D10, and HATMSC2F10 cells. Untreated cells were used as a control. Data represents mean ± SEM, *n* = 3

The pro-proliferative activity of HATMSC supernatants was checked using an in vitro model of chronic wound where human skin cell lines, fibroblasts (MSU-1.1), keratinocytes (HaCaT), and endothelial cells (HSkMEC.2), were seeded in 96-well plates in a serum-free medium under hypoxic conditions (1% O_2_) that mimic the chronic wound microenvironment, where often the blood supply is limited. First, to confirm the biological activity of native HATMSC supernatants, cells were treated with different percentages of HATMSC supernatant (0, 10, 25, 50, and 100%) in a serum-free medium. Then the metabolic activity of skin origin cells was measured at days 0, 1, 2, and 3 (Fig. 3a). Results showed an increase in all skin-derived cell survival in the presence of all tested HATMSC supernatants. For example, a significant increase (*p* < 0.001) in the metabolic activity of endothelial cells in the presence of supernatant from atMSC derived from the chronic wound patient (HATMSC1) was observed as early as day 1 and was maintained during the next 2 days. Similar effects were noted in the case of fibroblasts treated with HATMSC2 or HATMSC2D10 supernatant. Generally, with increased supernatant concentration, the proliferative activity of the supernatant was enhanced. The highest metabolic activity was observed following 3 days of treatment with 50% supernatant, while in the case of 100% supernatant, a sudden drop in metabolic activity was seen. Most probably this decrease stems from the fact that 100% supernatants, although rich in MSC-produced factors, are depleted of nutrients that were consumed by MSCs during supernatant production. Moreover, the proliferation activity of fibroblast was also verified following the addition of 50% primary HATMSC2 supernatant and compared to 50% of all HATMSC cell lines. The pro-proliferative activity of primary HATMSC2 supernatant was observed only at day 1 of the experiment, while at days 2 and 3, the proliferation of MSU-1.1 cells drastically decreased (Fig. 3b).

**Fig. 4:**
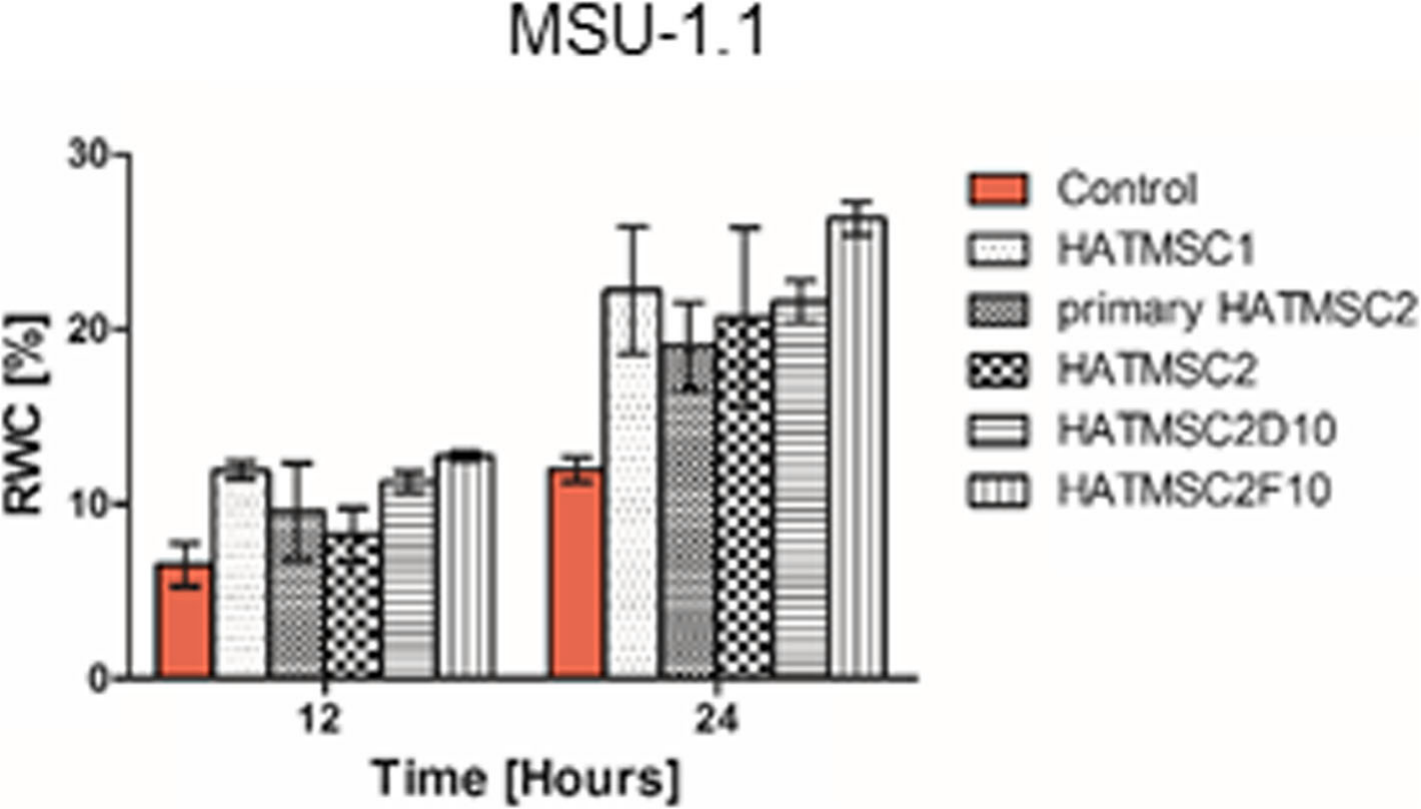
Migration activity of native HATMSC supernatants. Average relative wound closure of fibroblast (MSU-1.1) in the presence of 50% supernatants harvested from HATMSC1, primary HATMSC2, HATMSC2, HATMSC2D10, and HATMSC2F10 cells. Data represents mean ± SEM, *n* = 2

Furthermore, the influence of HATMSCs supernatants on fibroblast MSU-1.1 cell line migration was checked in wound healing assay. Fibroblast migration was investigated in the presence of 50% primary HATMSC2 supernatant and compared to 50% of all HATMSC cell lines supernatants. Obtained results showed that supernatants from all investigated HATMSCs increased migratory potential of fibroblasts (Fig. 4, Additional file 4).

### Native HATMSC supernatants promote in vitro angiogenesis of endothelial cells

**Fig. 5:**
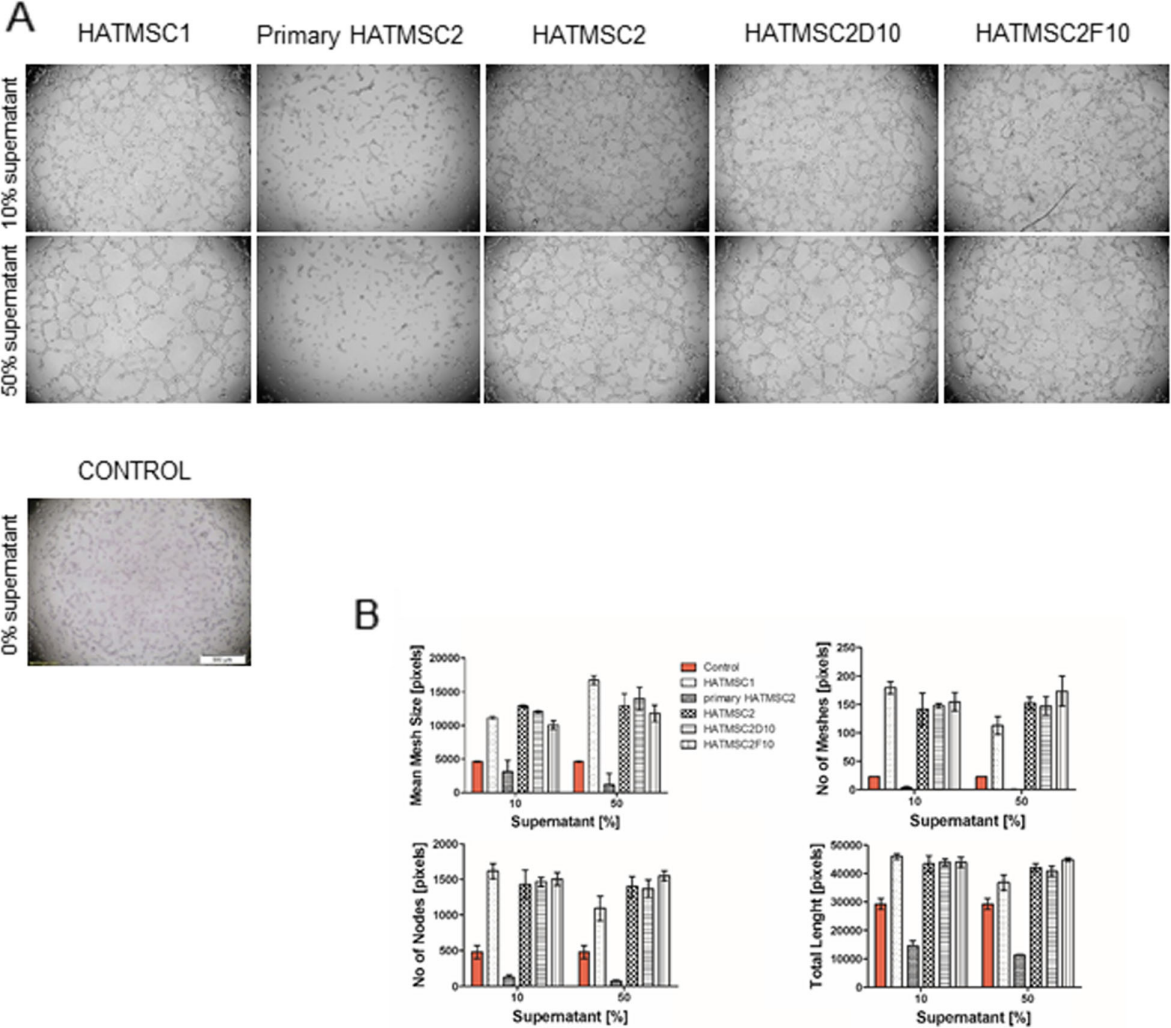
Pro-angiogenic activity of native HATMSC supernatants. **a** Representative images of in vitro angiogenesis of endothelial cells (HSkMEC.2) following 8 h of treatment with HATMSC supernatants including primary MSC supernatants group. **b** Quantification of mesh size, number of meshes, number of nodes, and total length of the tubes. Data represents mean ± SEM, *n* = 3

Since many angiogenesis-related factors were found in all HATMSC supernatants, their biological activity was investigated using the in vitro tube formation assay. Human skin endothelial cells (HSkMEC.2) were seeded into a 96-well plate coated with growth factor-reduced Matrigel. Cells were treated with 10 or 50% of supernatants from HATMSC1, primary HATMSC2, HATMSC2, HATMSC2D10, and HATMSC2F10. Cells cultured in DMEM without serum served as a control. Pictures were taken every 2 h to monitor pseudo-vessel formation. Results showed an increased number of pseudovessels in cultures treated with all HATMSC cell line supernatants, when compared to endothelial cells cultured in control medium alone (Fig. 5). Moreover, angiogenic properties were dose-dependent, e.g., 50% supernatants demonstrated stronger angiogenic potential than 10% (Fig. 5). No pro-angiogenic effect was observed when cells were treated with primary HATMSC2 cell supernatant.

### Concentrated HATMSC supernatants significantly increase proliferative potential of human skin origin cells subjected to in vitro model of chronic wound

**Fig. 6:**
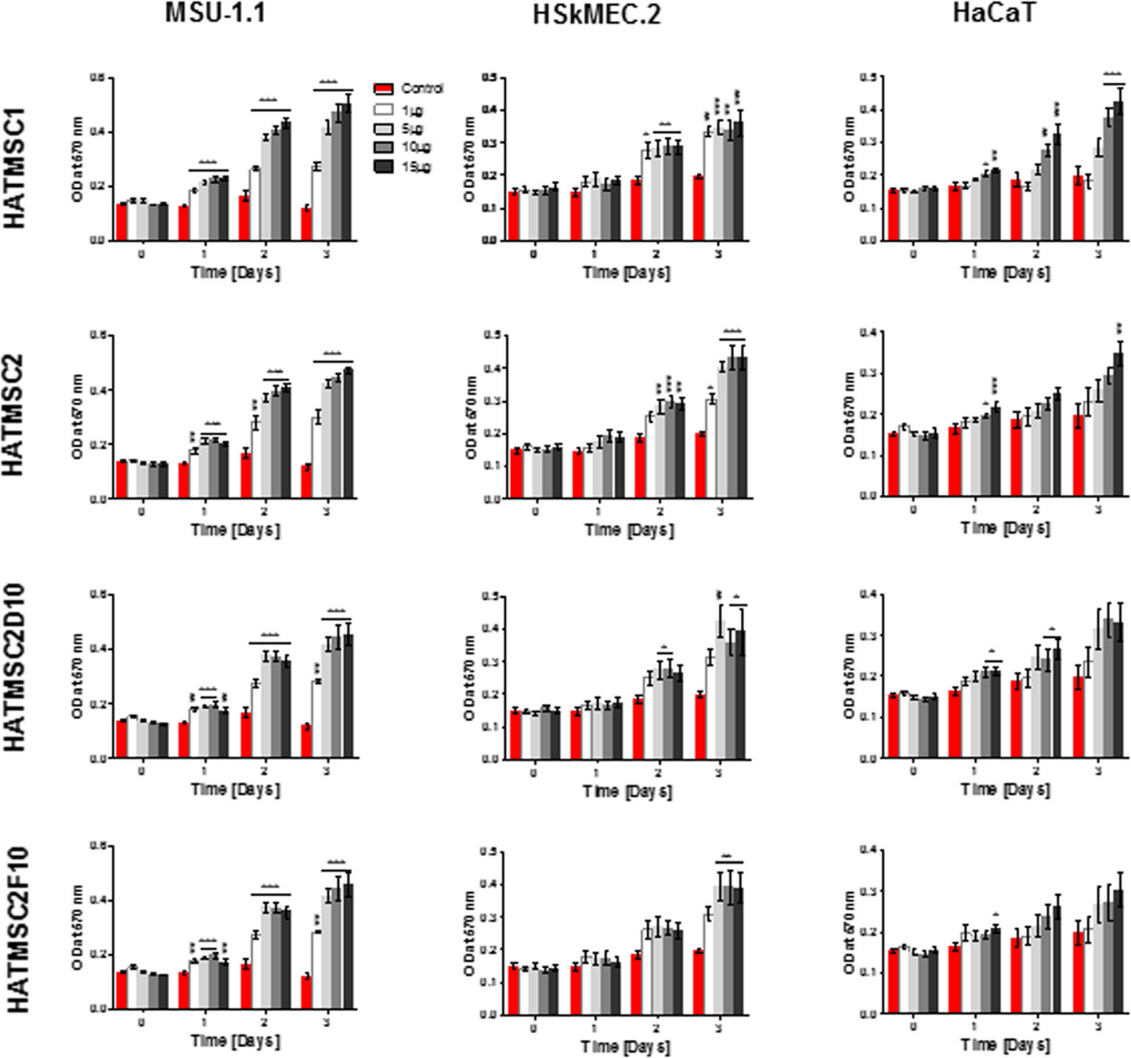
Proliferative activity of concentrated HATMSC supernatants in in vitro model of the chronic wound. Metabolic activity of MSU-1.1, HSkMEC.2, and HaCaT cells cultured in serum-free medium and 1% O_2_ was measured by MTT assay at days 0, 1, 2 and 3 following treatment with 1, 5, 10, and 15 μg of concentrated HATMSC supernatants. Untreated cells were used as a control. Data represents mean ± SEM, *n* = 3; *p < 0.05, **p < 0.01, ***p < 0.001

It was shown that native HATMSC supernatants contain important cytokines and growth factors for wound healing and thus promote skin cell survival in in vitro model of the chronic wound and induces in vitro angiogenesis of human skin endothelial cells. However, native supernatants are depleted from glucose and amino acids and contain metabolic products of atMSC. For this reason, native supernatants were concentrated using 3 kDa cut-off Amicon centrifuge tubes which resulted in approximately a 10-fold (v/v) increase in protein concentration. The biological activity of concentrated supernatants was confirmed as before, i.e., fibroblasts (MSU-1.1), keratinocytes (HaCaT), and endothelial cells (HSkMEC.2) were treated with 1, 5, 10, and 15 μg of total protein derived from concentrated supernatants and the metabolic activity measured at days 0, 1, 2, and 3 following treatment. As with native supernatants, the increase in cell proliferation using concentrated supernatants was observed as early as day 1 and was maintained during the next 2 days in the case of all tested supernatants and skin cell types (Fig. 6). All tested doses of supernatants revealed strong pro-proliferation properties, and an especially significant increase in metabolic activity was seen in cells treated with higher amounts of supernatant protein (5–15 μg, Fig. 6). In contrast, no effects of supernatant treatment were observed when the different types of skin origin cells were cultured under normoxia in DMEM with 10% serum (Additional file 3).

### Donor’s health condition did not influence the activity of HATMSC supernatants

**Fig. 7:**
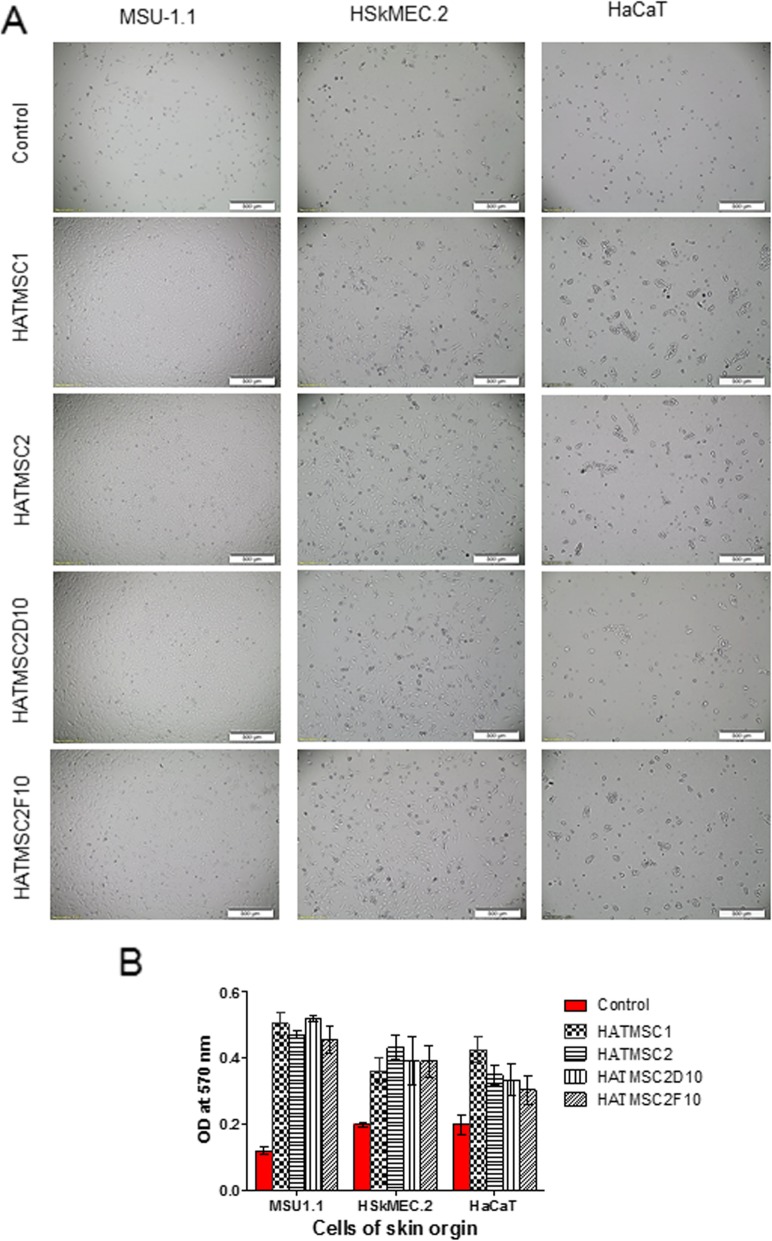
Comparison of pro-proliferative activity of supernatants derived from HATMSCs of chronic wound patients versus healthy donors. **a** Morphological changes of fibroblasts (MSU-1.1), endothelial cells (HSkMEC.2), and keratinocytes (HaCaT) following 3 days culture under 1% O_2_ and serum-free medium treated with 15 μg of concentrated HATMSC supernatants. **b** The effect of different origins of HATMSC supernatants on skin origin cell metabolic activity following 3 days culture in in vitro model of the chronic wound. Data represents mean ± SEM, *n* = 3

To determine whether the source of atMSCs influences their regenerative potential a comparative analysis between supernatants obtained from cells isolated from a patient suffering from venous stasis ulcer (HATMSC1) and a healthy donor (HATMSC2) was carried out. Additionally, two clones HATMSCD10 and HATMSCF10 established from the healthy donor cells were also compared. For this comparison, the proliferative effect of all four atMSC supernatants was checked in in vitro model of the chronic wound using fibroblasts, keratinocytes and endothelial cells. All types of cells were treated with concentrated atMSC supernatants using 15 μg of total protein per 100 μl of cell culture medium. Cell morphology and metabolic activity were assessed on the third day of treatment, when the differences between supernatant-treated and untreated control cells were the largest. Microscopic evaluation of cellular morphology showed that control cultures of all examined cells such as fibroblasts, keratinocytes, and endothelial cells contained a low number of total cells, where the majority of them exhibited symptoms of stress or appeared to be dying. In contrast, cultures supplemented with HATMSC supernatants contained high-density and healthy cells (Fig. 7a). Data from MTT assays supported these observations, demonstrating that cell viability was significantly increased in the presence of HATMSC supernatants, irrespective of their origin. A robust response to supernatant treatment was demonstrated by fibroblasts, where a fourfold increase in cell viability was observed. In keratinocytes and endothelial cell cultures, an addition of supernatants resulted in approximately a twofold increase in cell viability (Fig. 7b). In general, no substantial differences in pro-proliferative activity were observed when all established HATMSC cell lines were compared. Only in the case of keratinocytes, a slightly better regenerating effect was seen following treatment with supernatant produced by HATMSC1 line. On the other hand, in the case of endothelial cells, the highest increase in cell viability was seen while using HATMSC2-produced supernatant, although this did not reach statistical significance.

### Supernatant treatment of fibroblast subjected to in vitro chronic wound condition increased cell proliferation to a higher extent than co-culture with HATMSCs

**Fig. 8:**
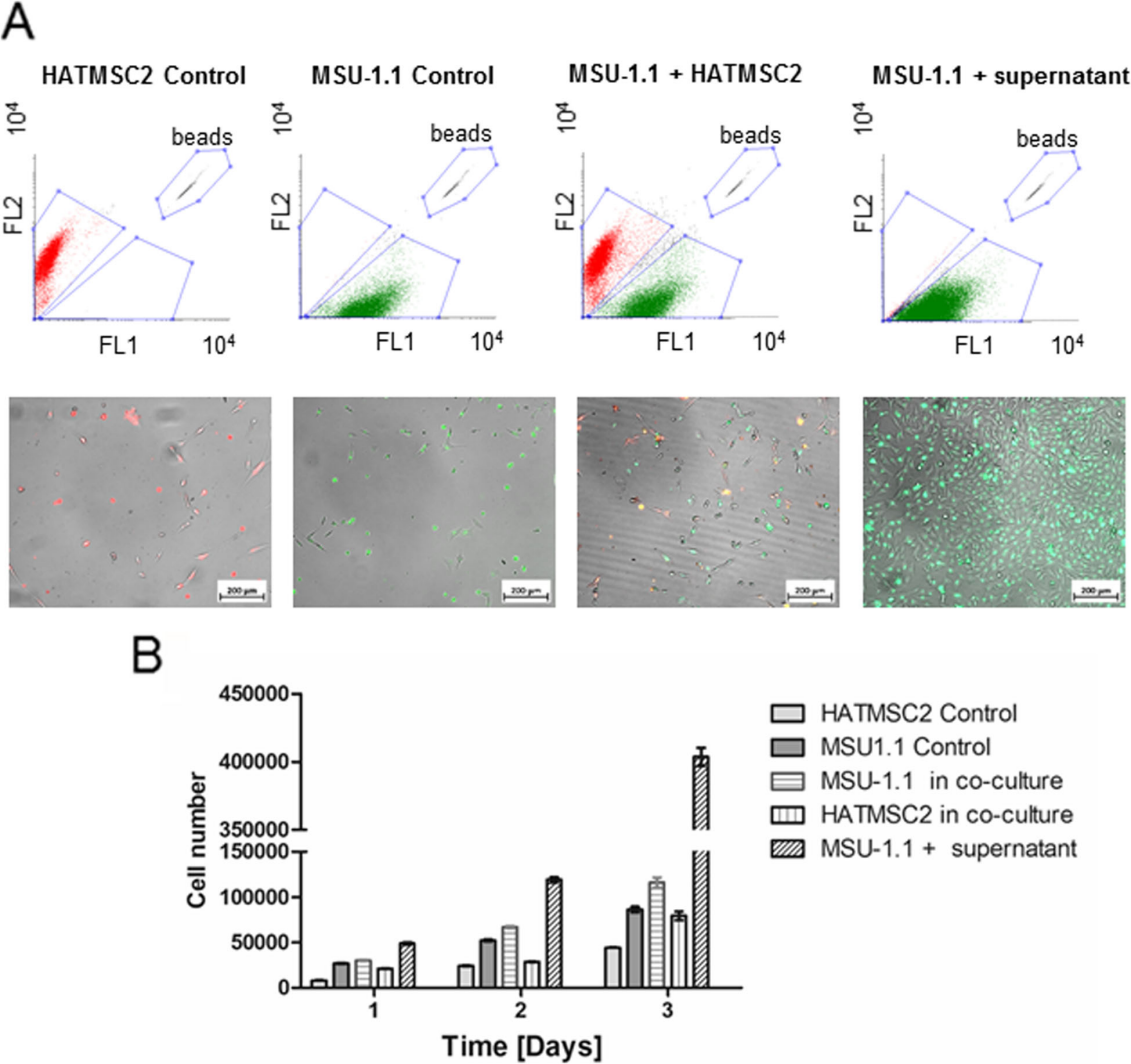
Comparison of pro-survival activity of HATMSC2 cells versus HATMSC2 supernatant on MSU-1.1 subjected to in vitro model of the chronic wound. **a** Representative dot plots and photos of fluorescently labeled cells HATMSC2 (red), MSU-1.1 (green), co-culture of MSU-1.1 with HATMSC2 and MSU-1.1 treated with HATMSC2 supernatant following 3 days culture. Dot plots showed a number of cells in FL1 channel for green fluorescence (MSU-1.1) and FL2 channel for red fluorescence (HATMSC2) in comparison to calibration beads. **b** Total cell number in tested groups at each time point. Data represents mean ± SD, *n* = 3

To compare the effect of HATMSC2 cells versus HATMSC2 supernatant on MSU-1.1 subjected to the in vitro model of chronic wounds, MSU-1.1 were co-cultured with HATMSC2 in a ratio 1:1 or were treated with the optimal dose of HATMSC2 supernatant (15 μg/100 μl). Flow cytometry analysis showed that the proliferation activity of MSU-1.1 cells was more effective in the supernatant-treated group than in the group co-cultured with HATMSC2 cells. This observation was also confirmed by fluorescence microscopy (Fig. 8a). Quantitative analysis of this experiment showed that co-culture with HATMSC2 increased the number of fibroblasts by approximately 20% in comparison to the control. However, following treatment with supernatant, an approximately 400% increase in fibroblasts proliferation was observed on the third day of the experiment (Fig. 8b). This difference may have resulted from concentrations of biologically active factors produced by HATMSC in co-culture being lower than in the concentrated supernatant derived from mono-cultures of the HATMSC cell line.

## Discussion

Cell-free therapy based on the application of the MSC secretome has gained a lot of research interest in recent years. This is due to a presence of a wide range of proteins and trophic factors with therapeutic potential released by the MSCs into culture supernatants [14, 19–21]. The synergistic effect of many factors that are naturally produced, glycosylated, and thereby much more stable than recombinant proteins, might provide more effective treatment. Employment of MSC-produced proteins instead of whole cells provides many advantages such as less invasiveness, increased safety, significant reduction of cost due to flexibility of GMP production, and the feasibility of long-term storage. Finally, the therapy based on active proteins instead of whole cells would be much more accessible for patients, without the requirement of hospitalization.

Bearing in mind the unsolved problem of chronic wound treatment, but also with respect to other degenerative diseases, in our study, we tested the regenerative potential of conditioned medium of newly developed human adipose tissue-derived mesenchymal stem cell lines. We began by immortalization of atMSC derived from a patient suffering from venous stasis ulcer and a healthy donor to check for potential therapeutic differences between cells obtained from patient versus healthy donor. It was shown previously that bone marrow MSC derived from healthy donor enhanced fibroblast migration more effectively than MSCs derived from chronic wound patients [22]. In our study, we did not observe significant differences in proliferation activity of supernatants produced by HATMSCs derived from chronic wound patient and healthy donor and profound therapeutic effect was observed in the case of all HATMSC cell line supernatants. This suggests that following immortalization, secretion activity of cells might be changed and supernatant content depends more on cell culture conditions rather than on the donor’s health condition. The second explanation may be that atMSCs, derived from a patient suffering from venous stasis ulcer, are not biologically affected, but their regenerative potential in vivo is insufficient due to the chronic disease. Isolated atMSCs from patients are able to secrete bioactive factors with pro-regenerative activity when cultured in vitro*,* which may be concentrated and applied to the patient to induce a pro-regenerative effect. However, donor-dependent differences in autologous MSC proliferation may limit this option for some patients [23]. In our study, when supernatants from primary HATMSC2 were used, no spectacular biological effect was observed compared to immortalized HATMSC cell lines. The reason for these could be that proliferation of primary cells is much slower than immortalized cells what may reduce the concentration of active factors in supernatants. Moreover, primary cells have a limited number of divisions and could change their paracrine activity with the number of passages. Therefore, a well-established MSC line/supernatant might be a better therapeutic alternative than autologous primary MSCs. Moreover, in contrast to primary cells, the usage of atMSC cell line enables a non-invasive but high-yield production of a unique bioactive mixture. Studies have shown that in the case of primary MSCs, the concentration of growth factors such as VEGF, EGF, and FGF in conditioned medium decreased rapidly between passages 3 and 15 [24]. An immortalized stable cell line should retain its secretion capability irrespective of the duration of culture and passage number. In comparison to supernatant from primary bone marrow MSC where maximum double increase in fibroblast proliferation was observed at the third day of treatment [25, 26], our HATMSC supernatants increased fibroblast survival approximately four times compared to serum-free control. Furthermore, the optimization of cell culture condition can increase the secretory activity of cells and thereby enrich the conditioned medium. In this study, atMSC supernatants were prepared under 1% of O_2_ since it was reported that hypoxia enhances growth factor production [27]. The developed HATMSC cell lines have retained MSC phenotype while gaining a high proliferation rate. Our in vivo experiment found no signs of tumorigenicity following the injection of immortalized cells into immuno-deficient mice, possibly increasing the therapeutic potential of our lines. However, possibly the most important feature of our lines is the capability to secrete a wide panel of angiogenic factors with potent biological activity that we have proven in a variety of in vitro functional assays. Although the secretory profile of human atMSC lines was discussed previously [28], the authors detected only two growth factors in the conditioned medium. Moreover, the biological activity of these factors has not been confirmed in vitro nor in vivo. In another study, where the effect of conditioned medium from primary human atMSCs was investigated on human skin cells, the paracrine profile was not analyzed at all and no increase in proliferation rate of fibroblast, keratinocyte or endothelial cells was observed [29]. Our in vitro studies confirmed profound pro-survival activity of all tested supernatants in chronic wound model; however, in the case of concentrated supernatants, this effect was observed at higher doses of total protein. When using native supernatants, the pro-survival effect was seen in the case of 50% supernatants, not 100%. We assume that 100% supernatants are depleted from nutrients, which were consumed by MSCs during supernatant production; therefore, pro-survival activity of such conditioned medium is reduced. This may also explain why other researchers who used native supernatants did not observed significant improvement in cell viability.

Finally, we have shown that in in vitro settings HATMSC supernatant treatment resulted in better fibroblast proliferation than in the case of co-culture with HATMSCs. This result suggests that therapy based on the MSC supernatant might lead to a more efficient therapeutic effect than MSC therapy alone. In contrast to MSC transplantation, the application of MSC supernatants allows for concentration of therapeutic proteins and at the same increases dose of therapeutic. Moreover, in contrast to cell transplantation, supernatant can be administered multiple times and, as such, it increases treatment efficiency.

Chronic or non-healing wounds are associated with a number of different pathological conditions such as diabetes, venous stasis, and chronic autoimmune diseases, and all of these diseases generally contribute to the generation of a hyper-inflammatory environment that further impairs the physiological healing processes [30]. It is known that MSCs release a soluble paracrine factor with pleiotropic immune regulatory activities. The use of HATMSC secretome for chronic wound treatment may inhibit the function of different immune cell subpopulations of the innate and adaptive immunity and thus diminish the inflammatory response. Moreover, tropic factors produced by HATMSCs promote human dermal fibroblast proliferation and may accelerate the re-epithelization of chronic wounds. Finally, pro-angiogenic growth factors secreted by HATMSCs modulate ischemic microenvironment of chronic wounds, augment neovasculogenesis, and accelerate wound closure via secretion of angiogenin, IL-8, VEGF, IGF-1, MCP-1, FGF, and other bioactive factors. Therefore, the biological activity of HATMSC secretome promotes angiogenesis and proliferation of keratinocytes and dermal fibroblasts leading to wound healing.

## Conclusions

In conclusion, the immortal HATMSC cell lines have a high proliferation rate without signs of tumorigenicity. Irrespective of the source of the atMSC cells (healthy donor versus chronic wound patient), they are capable of secreting a potent angiogenic cocktail that promotes human skin origin cell proliferation in an in vitro chronic wound model. Based on available literature and our present results, HATMSC supernatants seem to be more efficient in pro-regenerative activity in vitro than other conditioned media produced by primary or immortalized MSCs and therefore have high clinical implementation potential for use as a source of growth factors and cytokines for multiple regenerative medicine applications.

## Supplementary information

**Additional file 1.** Proliferation profile of HATMSC1, HATMSC2, HATMSC2D10 and HATMSC2F10 cells. Cells were seeded in triplicate into a 96-well plate at a density of 2.0 x 10^3^ cells per well in 100 μl of DMEM+10% serum, under normoxic (LH panel) or hypoxic (1% O_2_) conditions (RH panel). The metabolic activity of cells was measured using an MTT assay at days 0, 1, 2 and 3. Line graphs represent the mean value ± SD of three independent experiments.

**Additional file 2.** Evaluation of tumorigenicity in NOD SCID mice following subcutaneous injection of HATMSC2 cells. Haematoxylin and Eosine staining of paraffin-fixed sections of different tissue collected from animals following 16 weeks after cell injection showed no evidence of pathologic changes or tumor formation. Arrows indicate clusters of hemosiderin in the spleen.

**Additional file 3. **Influence of concentrated HATMSCs supernatants on metabolic activity of skin origin cells cultured under standard conditions. 2.0 x 10^3^ of MSU-1.1, HSkMEC.2 and HaCaT were seeded in 10% FBS in DMEM in a well of 96-well plate in triplicates. Cells were treated with 1, 5, 10 and 15 μg of concentrated HATMSC supernatants and were incubated under normoxic conditions (5% CO_2_, 37 °C) for 0, 1, 2 and 3 days. Cell metabolic activity was measured at each time point by MTT assay. Data represents mean ± SEM, *n* = 3.

**Additional file 4.** Migration activity of native HATMSC supernatants. MSU-1.1 cell migration activity was investigated at 37 °C in an incubation chamber (PeCon GmbH, Erbach, Germany) with 1%O_2_, 5%CO_2_ mounted on an Axio Observer inverted microscope equipped with a dry 5x objective (Zeiss, Gottingen, Germany). The movement of the cells was time-lapse recorded for 44 h at intervals of 2 h using Zen 2.6 Blue Edition Software (Zeiss, Gottingen, Germany) as 6 separate movies (one for each supernatant and control).

## Data Availability

All data generated or analyzed during this study are included in this published article.

## Abbreviations

atMSC: Adipose tissue-derived mesenchymal stem cell
DMEM: Dulbecco’s modified Eagle’s medium
EGF: Epidermal growth factor
FBS: Fetal bovine serum
FC: Flow cytometry
FGF: Fibroblast growth factor
GRO: Growth-regulated oncogene
HATMSC: Human adipose tissue mesenchymal stem cell lines
HS: Human serum
IGF: Insulin-like growth factor
IL: Interleukin
MCP-1: Macrophage chemoattractant protein-1
MMP: Matrix metalloproteinase
MTT: (3-(4,5-Dimethylthiazol-2-yl)-2,5-Diphenyltetrazolium Bromide)
PE: Phycoerythrin
RANTES: Regulated on Activation, Normal T Cell Expressed and Secreted
TIMP: Tissue inhibitor of metalloproteinase
VEGF: Vascular endothelial growth factor
