# Supplementary figures and images for: Can supernatant from immortalized adipose tissue MSC replace cell therapy? An in vitro study in chronic wounds model
Source: Stem Cell Res Ther. 2020 Jan 21;11:29. doi: 10.1186/s13287-020-1558-5 (PMC6975034; doi:10.1186/s13287-020-1558-5)

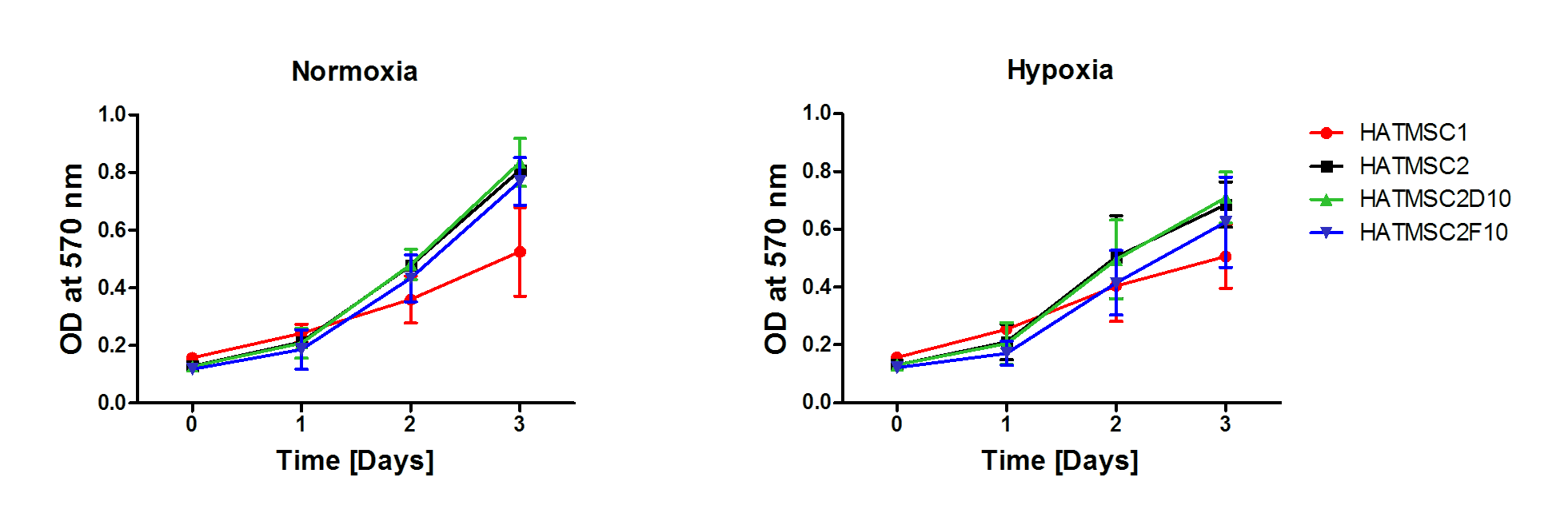

Supplement: Supplementary file 1 — Additional file 1. Proliferation profile of HATMSC1, HATMSC2, HATMSC2D10 and HATMSC2F10 cells. Cells were seeded in triplicate into a 96-well plate at a density of 2.0 x 10^3^ cells per well in 100 μl of DMEM+10% serum, under normoxic (LH panel) or hypoxic (1% O2) conditions (RH panel). The metabolic activity of cells was measured using an MTT assay at days 0, 1, 2 and 3. Line graphs represent the mean value ± SD of three independent experiments. [file 13287_2020_1558_MOESM1_ESM.tif]

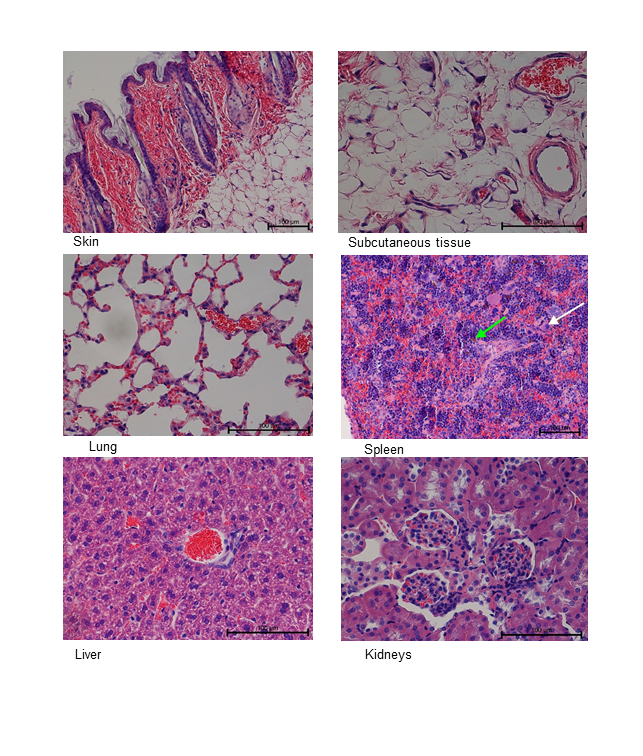

Supplement: Supplementary file 2 — Additional file 2. Evaluation of tumorigenicity in NOD SCID mice following subcutaneous injection of HATMSC2 cells. Haematoxylin and Eosine staining of paraffin-fixed sections of different tissue collected from animals following 16 weeks after cell injection showed no evidence of pathologic changes or tumor formation. Arrows indicate clusters of hemosiderin in the spleen. [file 13287_2020_1558_MOESM2_ESM.tif]

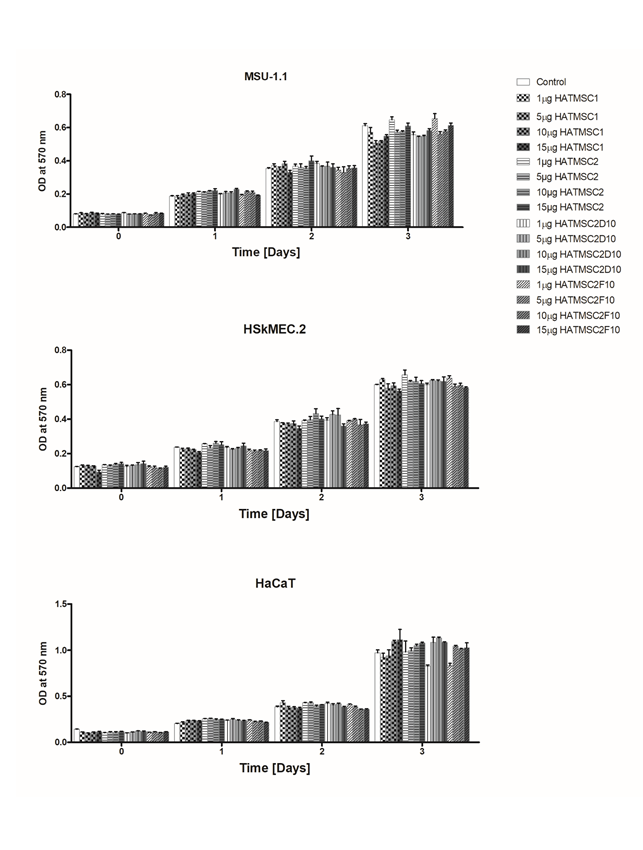

Supplement: Supplementary file 3 — Additional file 3. Influence of concentrated HATMSCs supernatants on metabolic activity of skin origin cells cultured under standard conditions. 2.0 x 10^3^ of MSU-1.1, HSkMEC.2 and HaCaT were seeded in 10% FBS in DMEM in a well of 96-well plate in triplicates. Cells were treated with 1, 5, 10 and 15 μg of concentrated HATMSC supernatants and were incubated under normoxic conditions (5% CO2, 37 °C) for 0, 1, 2 and 3 days. Cell metabolic activity was measured at each time point by MTT assay. Data represents mean ± SEM, n = 3. [file 13287_2020_1558_MOESM3_ESM.tif]
